# Astrocyte-Derived Vascular Endothelial Growth Factor Stabilizes Vessels in the Developing Retinal Vasculature

**DOI:** 10.1371/journal.pone.0011863

**Published:** 2010-07-29

**Authors:** Andrew Scott, Michael B. Powner, Pranita Gandhi, Claire Clarkin, David H. Gutmann, Randall S. Johnson, Napoleone Ferrara, Marcus Fruttiger

**Affiliations:** 1 UCL Institute of Ophthalmology, University College London, London, United Kingdom; 2 Division of Reproduction and Endocrinology, King's College London, London, United Kingdom; 3 Department of Neurology, Washington University, St. Louis, Missouri, United States of America; 4 University of California San Diego, La Jolla, California, United States of America; 5 Genentech Inc., South San Francisco, California, United States of America; University of Reading, United Kingdom

## Abstract

Vascular endothelial growth factor (VEGF) plays a critical role in normal development as well as retinal vasculature disease. During retinal vascularization, VEGF is most strongly expressed by not yet vascularized retinal astrocytes, but also by retinal astrocytes within the developing vascular plexus, suggesting a role for retinal astrocyte-derived VEGF in angiogenesis and vessel network maturation. To test the role of astrocyte-derived VEGF, we used Cre-lox technology in mice to delete VEGF in retinal astrocytes during development. Surprisingly, this only had a minor impact on retinal vasculature development, with only small decreases in plexus spreading, endothelial cell proliferation and survival observed. In contrast, astrocyte VEGF deletion had more pronounced effects on hyperoxia-induced vaso-obliteration and led to the regression of smooth muscle cell-coated radial arteries and veins, which are usually resistant to the vessel-collapsing effects of hyperoxia. These results suggest that VEGF production from retinal astrocytes is relatively dispensable during development, but performs vessel stabilizing functions in the retinal vasculature and might be relevant for retinopathy of prematurity in humans.

## Introduction

Retinal astrocytes play an important role in the development of the mammalian retinal vasculature. They invade the retina from the optic nerve head as a proliferating population of cells and spread across the inner surface of the retina, creating a template for the developing retinal vasculature which follows in their wake [1]–[4]. There is a tight correlation between the presence of retinal astrocytes and the retinal vasculature. In animals with only partially vascularized retinas, such as rabbit and horse, retinal astrocytes are absent from the avascular regions of the retina [5], [6]. Furthermore, in primates, retinal astrocytes are absent from the foveal avascular zone [7], [8].

Numerous studies have shown that during retinal vascularization retinal astrocytes produce high levels of *Vegf* mRNA in the not yet vascularized peripheral portion of the retina [9]–[11]. This part of the retina is experiencing physiological hypoxia during development [9], increasing VEGF transcription possibly via hypoxia inducible factors (HIFs) or *Vegf* mRNA stabilization [12], [13]. This differential expression of VEGF - high in the periphery and low in the centre - might lead to a gradient providing a directional stimulus for retinal vascularization. Furthermore, the development of the retinal vasculature can be accelerated and delayed by intra ocular injection of inhibitors or activators of VEGF signalling [14]. VEGF is therefore considered a likely mediator of astrocyte-vessel interactions during the outgrowth of the retinal vasculature.

Apart from driving angiogenesis, VEGF may also influence vessel remodelling, stabilization and differentiation during vascular plexus maturation. The stability of vessels plays an important role in the pathogenesis of retinopathy of prematurity (ROP), where exposure of premature infants to therapeutic hyperoxia can cause vaso-obliteration in the developing retinal vasculature [15]. Many in vitro studies have demonstrated that VEGF can act as a mitogen and as a survival factor for endothelial cells [16]–[19]. Evidence from in vivo models further supports this. For instance, it is likely that the strong vaso-obliterative effects of hyperoxia in the retina are at least in parts mediated via the suppression of VEGF expression. Exposing mouse pups to a 75% oxygen atmosphere from postnatal day 7 (P7) to P12 obliterates capillaries in the centre of the retina [20]. This correlates with a reduction of VEGF expression in the same retinal region and can be prevented by intra vitreal injection of VEGF [1], [21]. Furthermore, high oxygen levels in arterial blood are likely to be responsible for suppression of *Vegf* mRNA near arteries and consequently for the formation of so-called capillary free spaces along arteries [22]. These capillary free spaces widen when mice are exposed to hyperoxia and disappear when the pups are raised under hypoxic conditions. These changes correlate with decreased and increased VEGF expression respectively [21], [23], further supporting the notion that VEGF expression within the vascular plexus influences vessel survival and consequently network topology.

It has been shown that VEGF secreted from pericytes can also contribute to vessel stabilization [24], and vessels lacking mature pericytes are prone to regression in the hyperoxia model [25]. Stabilization of blood vessels is based on a endothelial cell-pericyte interactions [26], which clearly plays an important role in the remodelling and shaping of the retinal vasculature. This is further illustrated by the fact that hyperoxia exposure spares the larger, more mature arteries and veins that are radially projecting from the centre to the periphery. Also, in more mature animals (about 3 weeks old) the retinal vasculature is no longer sensitive to hyperoxia-induced vaso-obliteration [27].

However, it is less clear to which degree retinal astrocytes and pericytes contribute to vessel stabilization and how important VEGF secretion from these cells is during the critical period of network maturation in the first 2–3 weeks after birth. We therefore investigated the role of astrocyte-derived VEGF in retinal vascular development by genetically abolishing VEGF expression using the Cre-lox system. Surprisingly, this only had a minor impact on the physiological development of the retinal vasculature. However, under hyperoxic conditions deletion of astrocyte-derived VEGF led to a pronounced destabilization of the retinal vasculature.

## Results

### Retinal astrocyte-derived VEGF is not required for retinal vascularization

**Figure 1:**
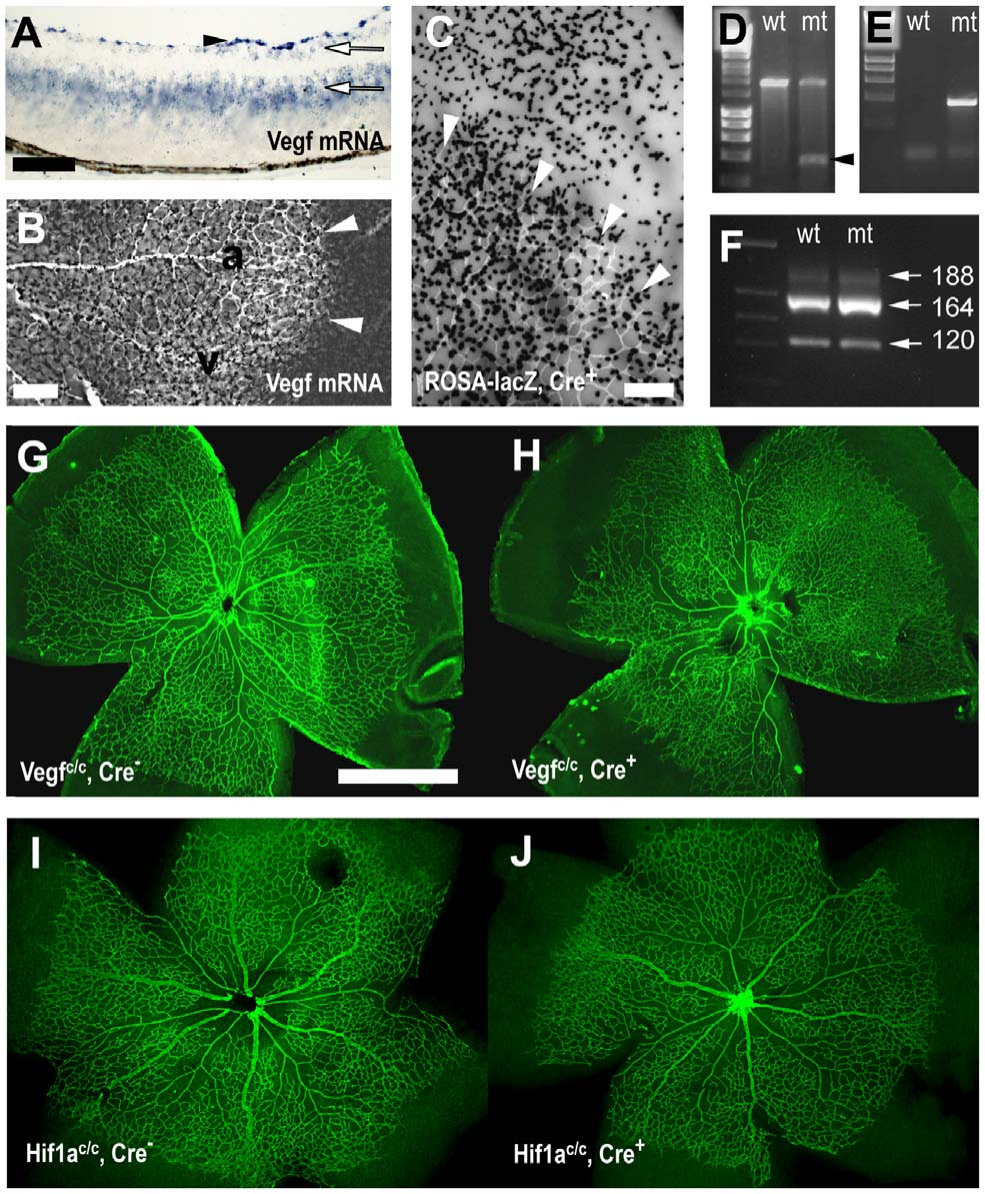
Astrocyte specific deletion of VEGF. (A, B) In situ hybridisation showed *Vegf* mRNA expression in a retinal cross-section (A) and a retinal whole mount (B) at P5. (A) *Vegf* mRNA was most strongly expressed in retinal astrocytes (arrowhead), and weakly in retinal ganglion cells and the inner nuclear layer (arrows). (B) Retinal astrocytes expressed VEGF strongest distally to the edge of growing vascular plexus (white arrowheads), at intermediate levels around veins (v) and weakest along arteries (a). (C) Transgenic mice expressing Cre recombinase in retinal astrocytes (*Gfap-Cre* mice) showed in a lacZ reporter strain recombination activity (black X-gal stain in C) in retinal astrocytes proximally and distally to the growing edge of the vascular plexus (arrowheads in C). (D, E) Cre recombination in *Vegf* ^c/c^ mice, crossed with *Gfap-Cre* mice, was demonstrated by PCR across the deleted region in genomic DNA from retinas in Cre positive (mt) and Cre negative (wt) animals (D). PCR on tail DNA identified Cre negative and positive animals (E). (F) PCR on cDNA from retinal mRNA showed unchanged VEGF isoform ratios in control and mutant animals. Astrocyte deletion of VEGF led to subtle abnormalities in the P5 retinal vasculature (stained with an anti-claudin 5 antibody G–J) in Cre positive animals (H) compared to wild type litter mates (G). In contrast, deletion of HIF1α in retinal astrocytes caused no such abnormalities (I–J). Scale bars are 100µm in A, 200µm in B, C and 1000µm in G.

In the developing retina *Vegf* mRNA is expressed by retinal astrocytes, retinal ganglion cells and cells in the inner nuclear layer (Fig. 1A) [7], [28]–[30]. Retinal astrocytes display the strongest *Vegf* mRNA expression, with particularly high levels distally to the growing vascular plexus, where the tissue is still avascular and hypoxic (Fig. 1B). To test the role of retinal astrocyte-derived VEGF, we employed the Cre-lox system. Astrocytes were targeted using a mouse strain that expresses Cre recombinase under the control of the glial fibrillary acidic protein (*Gfap*) promoter (*Gfap-Cre* mice) [31]. Retinal astrocytes-specific Cre recombination activity was confirmed in the ROSA-lacZ reporter strain [32], which demonstrated lacZ expression in retinal astrocytes within the vascular plexus but also in astrocytes in the peripheral, not yet vascularized portion of the retina (Fig. 1C).

Retinal astrocyte deletion of VEGF was achieved by breeding the *Gfap-Cre* allele into a homozygous background containing loxP sites flanking exon 3 of the *Vegfa* gene (*Vegf* ^c/c^ mice) [33]. Offspring containing the *Gfap-Cre* allele was compared against littermates without the transgene. Cre recombinase activity in *Gfap-Cre* positive animals was confirmed by PCR of genomic DNA isolated from the retina with primers spanning a 2.1kb region across exon 3, which demonstrated deletion of a 1.3kb region in the *Vegfa* gene with the appearance of an additional shorter band of 800bp in Cre-positive animals (Fig. 1D, E). The larger 2.1kb band remains in transgenic animals because VEGF is not only expressed in retinal astrocytes but also in other retinal cells, such as neurons and Müller cells, which are not affected by *Gfap-Cre* strain-mediated excision.

It is possible that different cells in the retina express different VEGF isoforms [34], [35]. We therefore tested whether astrocyte VEGF deletion can change the VEGF isoform ratios. RT-PCR on mRNA isolated from P5 retinas revealed that VEGF_164_ was the dominant isoform in the retina, whereas VEGF_120_ was expressed at lower levels and VEGF_188_ was barely detectable. Importantly, this was found in both wild type and astrocyte VEGF knock-out animals (Fig. 1F), demonstrating that VEGF isoforms ratios are not changed.

In order to assess the effects of astrocyte deletion of VEGF in the retina, we studied the retinal vasculature at P5. Surprisingly, development of the retinal vasculature was almost normal apart from subtle abnormalities. In Cre-positive mutant mice, the vascular network seemed slightly irregular and less developed than in their wild type counter parts (Fig. 1G, H). Since hypoxia can regulate VEGF transcription via hypoxia-inducible factor 1 alpha (HIF1α) we also studied animals with astrocyte deletion of HIF1α. To this end, the *Gfap-Cre* strain was crossed into a background containing homozygous floxed *Hif1a* alleles [36]. This did not lead to the same abnormalities observed following VEGF deletion, and had no apparent effect on the retinal vasculature whatsoever (Fig. 1I, J), demonstrating that HIF1α does not contribute significantly to the regulation of VEGF in the retinal astrocytes. The lack of any noticeable phenotype after HIF1α deletion also served as a negative control against possible non-specific effects of the *Gfap-Cre* transgene.

### Delayed angiogenesis

**Figure 2:**
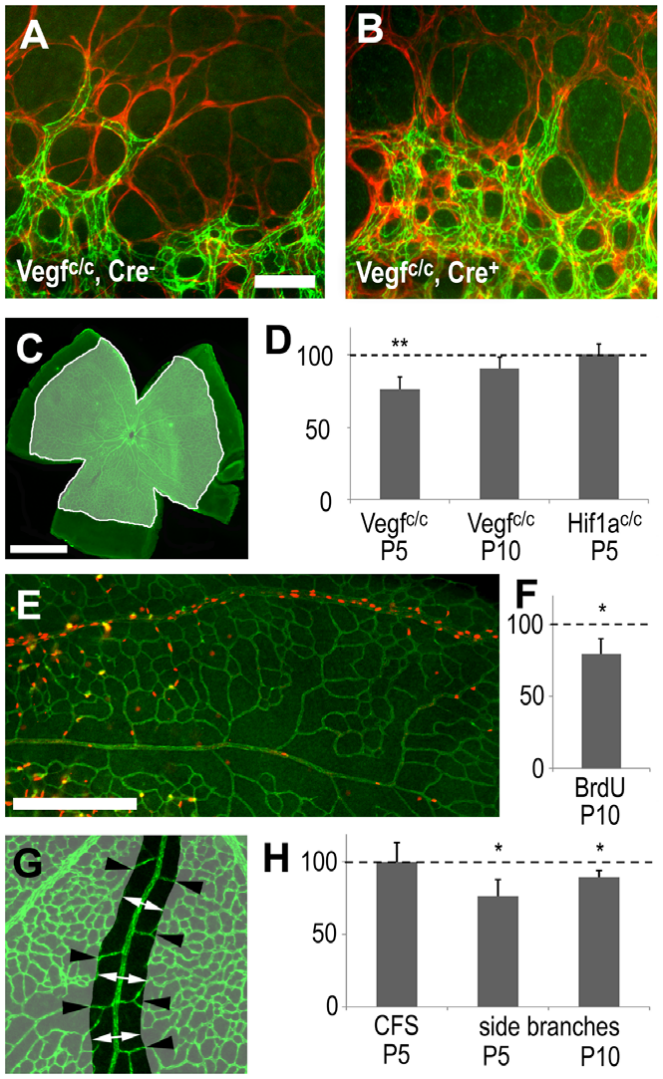
Effects of astrocyte-derived VEGF on retinal vascular development. Immunohistochemistry, visualizing endothelial cells (claudin 5, green in A, B, C, E, G) and retinal astrocytes (GFAP, red in A, B), shows that angiogenic sprouting at the leading edge of the growing vascular plexus appears normal in control (A) and mutant (B) animals lacking astrocyte derived VEGF. (C, D) Measurement of the surface area of the retinal vasculature (C) showed significantly reduced spreading in *Vegf* ^c/c^ mutants at P5 but not at P10 and not in P5 *Hif1a* ^c/c^ mutants at P5. (E) AT P10 proliferation (anti-BrdU in red) occurs predominantly in veins and is reduced in animals lacking astrocyte-derived VEGF in comparison to littermate controls. (G–H) Endothelial cell survival near arteries was assessed by measuring the width of the capillary free zone (CFS, white arrows) and the number of artery side branches (black arrowheads). (H) In mutant animals CFS width was not affected but the number of side branches was reduced. Scale bars are 50µm in A, 1000µm in C and 200µm in G; * is p<0.05 and ** is p<0.01.

The pronounced expression of VEGF in peripheral astrocytes (in the not yet vascularized part of the retina) has been linked to sprouting angiogenesis at the leading edge of the expanding vascular plexus. We therefore assessed whether the absence of VEGF in astrocytes affected the morphology of angiogenic sprouts at the leading edged of the growing vascular plexus, but found no noticeable differences (Fig. 2A, B). To further investigate effects on vascular sprouting, the surface area covered by the retinal vasculature was measured (Fig. 2C). Quantification of 6 separate litters at P5 revealed that average spreading of the vessel network in the mutant animals was reduced by 24% (p = 0.004) (Fig. 2D). This represents a subtle, but significant, delay of angiogenesis. However, at P10, this difference was reduced to 10%, and was no longer statistically significant (p = 0.053). These findings suggest that the observed retinal vasculature impairment was transient and that the delay in angiogenesis was eventually overcome (Fig. 2D). No delay was observed after astrocyte HIF1*α* deletion (Fig. 2D), confirming the lack of phenotype shown above (Fig. 1I, J).

### Effect of astrocyte-derived VEGF on endothelial cell proliferation and survival

Although vascularization causes retinal astrocytes to downregulate *Vegf* mRNA, the in situ hybridization signal was still detectable within the vascular plexus, particularly along veins (Fig. 1B). It is possible that this VEGF expression influences the proliferation and survival of endothelial cells as the vascular network undergoes remodelling and maturation. For instance, the relatively high levels of *Vegf* mRNA near veins correlate with high proliferation rates in developing veins (Fig. 2E). To assess a possible effect of astrocyte-derived VEGF, we quantified the number of BrdU-positive nuclei in veins in P10 animals. At this age, the vascular plexus undergoes extensive remodelling, and proliferation rates were noted between veins and arteries. In our VEGF-deleted animals, the number of labelled cells was reduced by 20% (p = 0.027) compared to control animals, again suggesting a small but significant influence of astrocyte-derived VEGF (Fig. 2F).

In order to establish whether astrocyte-derived VEGF can also influence endothelial cell survival in the developing retinal vasculature, we analyzed network morphology around arteries. VEGF expression is naturally very low in these regions and abolishing astrocyte-derived VEGF is therefore likely to have the biggest impact on endothelial cell survival near arteries. We measured the width of capillary free spaces around arteries, but found no difference between VEGF-deleted and control animals (Fig. 2G, H). We then counted the number of primary artery side branches, and found a reduction at P5 (24%, p = 0.011) and at P10 (11%, p = 0.014), demonstrating that astrocyte-derived VEGF contributes to endothelial cell survival within the capillary-free zones around arteries (Fig. 2 G, H).

### Astrocyte-derived VEGF protects vessels from oxygen-induced obliteration

**Figure 3:**
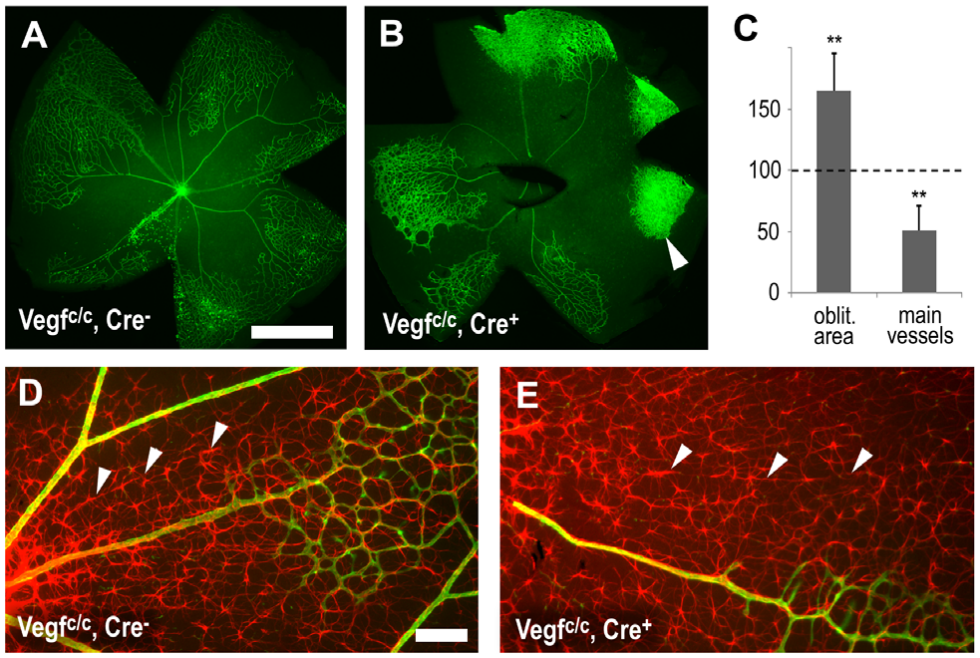
Astrocyte-derived VEGF protects vessels from hyperoxia. After hyperoxia exposure from P7–12 immunohistochemistry was used to visualize vessels with isolectin B4 (green, A, B), collagen IV (green, D, E) and retinal astrocytes (GFAP, red, D, E). (A–C) Deletion of astrocyte specific VEGF increased the vaso-obliterated area and led to decreased survival of radial arteries and veins. In some instances this lead to non-perfused, hyperproliferating capillary beds in the periphery (arrowhead in B). (D, E) Retinal astrocyte survival was not affected at this age but in areas of capillary loss astrocytes re-aligned with nerve bundles (arrowheads D, E). Scale bars are 100µm in A and 100µm in D; ** is p<0.01.

Since astrocyte-derived VEGF increases endothelial cell survival in the relatively high oxygen environment near arteries, we decided to test whether this is also true under more extreme circumstances. To this end, P7 pups were exposed to 75% oxygen for 5 days, which obliterates capillaries around arteries and in the centre of the retina. In animals lacking astrocyte-derived VEGF, the area where capillaries were obliterated was dramatically increased (65%, p = 0.003, Fig. 3A–C). Furthermore, the usually stable main arteries and veins showed an increased tendency to collapse causing a reduction of vessels extending from the optic nerve (49%, p = 0.004, Fig. 3A–C). In some instances this led to isolated, non-perfused patches of capillaries in the periphery (arrowhead Fig. 3B). Since VEGF levels are not suppressed by the 75% oxygen atmosphere in this region of the retina [21], vessels survive. However, they hyperproliferate and normal network morphology is lost, demonstrating important stabilizing effects of vascular perfusion on vascular network development.

It has been reported that hyperoxia can induce retinal astrocyte death [37]–[39]. To determine whether astrocyte cell death contributes to the effects observed in our mutant animals, we visualized retinal astrocytes at P12 (using an anti-GFAP antibody). The astrocytes had similar morphologies and densities in control and mutant animals (Fig. 3D, E). Quantification of cell numbers also revealed no differences (not shown). Interestingly, while mouse retinal astrocytes normally show a strong spatial interaction with blood vessels, following hyperoxia-induced vessel-obliteration, retinal astrocytes re-aligned themselves radially with what appear to be axon fibre bundles (arrowheads Fig. 3D, E).

**Figure 4:**
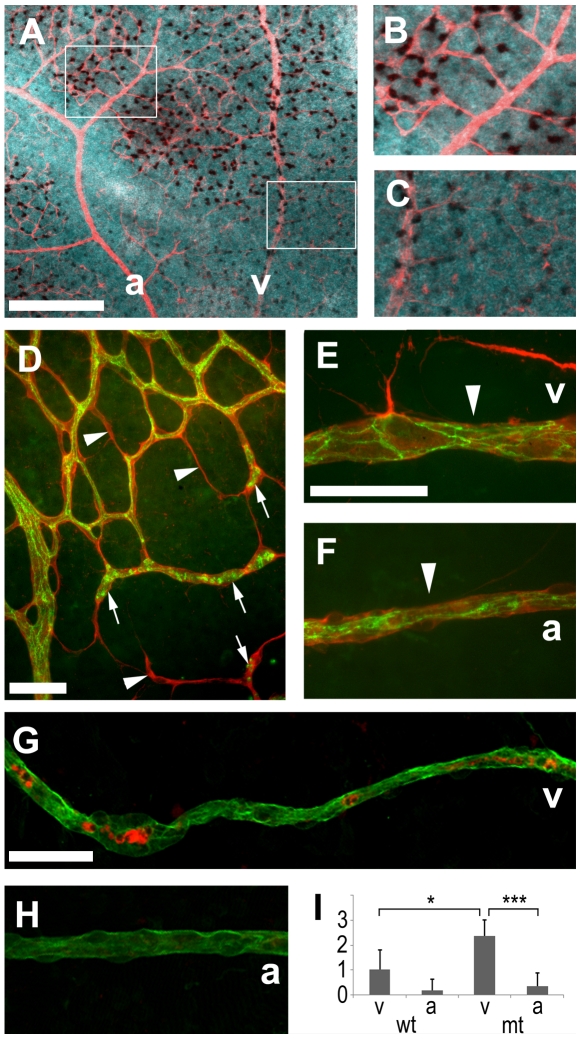
Vessel degeneration after one day of hyperoxia (P7–8). (A–C) In situ hybridization showed that *Vegf* mRNA was detectable within the vascular network (stained with anti-collagen IV, red) but strongly reduced around arteries. Immunohistochemistry with anti-collagen IV (red D–F, green G, H), anti-claudin 5 (green D–F) and active caspase 3 (red G, H) revealed dying vessels. Empty basement membrane sleeves (arrows D) and isolated claudin 5 positive clumps were indicative of a regressing capillary network and local narrowing of artery and vein profiles (arrowheads E, F) suggested blood flow reductions. (G–I) Astrocyte-specific VEGF deletion increased radial vessel degeneration and affected veins more strongly than arteries at this early time point. Scale bars are 200 µm in A and 50 µm in D–G; * is p<0.05 and *** is p<0.001.

In order to gain more insight into the process of vessels-obliteration, we also studied animals after 24 hours of hyperoxia exposure at P8. *Vegf* mRNA was suppressed most strongly around arteries, but still detectable throughout the network (Fig. 4A–C). Immunohistochemistry with antibodies against collagen IV and claudin 5 (labelling basement membranes and endothelial cell junctions respectively, Fig. 4D–F) was used to visualize capillaries at different stages of obliteration. In some instances, only thin basement membrane sleeves, devoid of endothelial cells, could be observed (arrowheads Fig. 4D). Other vessels contained claudin 5 positive clumps (most likely dying endothelial cells) and were isolated from the rest of the network (arrows Fig. 4D). The earliest abnormality displayed by radial veins and arteries were local reductions in vessel diameter with seemingly still intact endothelial junctions (Fig. 4E, F). Active caspase-3-positive reactivity was found inside vessels with degenerating morphology, but not in vessels with normal appearance (Fig. 4G, H), indicating that endothelial cells undergo apoptosis within collapsing blood vessels. In wild type animals, we found typically one degenerating vein per retina and only one degenerating artery every five retinas (Fig. 4I). In animals that lacked astrocyte-derived VEGF, this ratio between veins and arteries roughly persisted (6∶1, p = 0.001) but the overall frequency of visibly degenerating vessels more than doubled. This was particularly obvious with veins, which degenerated 2.4 times more often (p = 0.019) in mutant animals (Fig. 4I). These results suggest that at this stage of retinal vascular development veins are more vulnerable to hyperoxia and that astrocyte-derived VEGF has a stabilizing and protecting influence on them.

## Discussion

Our experiments have demonstrated that the role retinal astrocytes play in retinal vascularization is mediated only in a small part by VEGF. The most likely explanation for the minor effects of astrocyte VEGF deletion on retinal angiogenesis is compensation of VEGF production by other cells, such as neurons. In comparison to retinal astrocytes, RGCs and cells in the inner nuclear layer display a much weaker in situ hybridization signal for *Vegf* mRNA, but because these low expressing cells are more abundant, VEGF is likely to be produced in sufficient quantities to allow for almost normal vascularization. It is likely that there are other aspects of retinal astrocytes that involve these cells critically in retinal vascularization. For instance, it has been suggested that retinal astrocytes mediate extracellular assembly of fibronectin matrices required for vessel growth [40]. It is also possible that they provide not yet identified factors that are required for retinal angiogenesis.

Nevertheless, retinal vascularization was not entirely unaffected after astrocyte VEGF deletion. Speed of angiogenesis, endothelial cell proliferation and survival were all subtly but reproducibly reduced. Every one of these features suggests that there is reduced VEGF activity in the retina during development, which is consistent with our experimental design that targeted only a subpopulation of cells in the retina. Interestingly, deletion of HIF1α had no effect on the development of the retinal vasculature, suggesting that in retinal astrocytes, the primary regulation of VEGF expression is HIF1α-independent. In concordance with that, in mice lacking the hypoxia response element in the VEGF promoter the retinal vasculature develops normally [41]. Alternatively, VEGF might also be regulated via the stabilization of its mRNA under hypoxic conditions [12]. In fact, it has been shown that regulation of VEGF production via this mechanism is more potent in comparison to transcriptional control [42].

Compared to the relatively subtle effects on the speed of retinal angiogenesis and endothelial cell proliferation, astrocyte VEGF deletion had more pronounced consequences on vessel stability. Although, the width of capillary free spaces was unchanged, the number of artery side branches was clearly reduced. It therefore appears that during normal development astrocyte-derived VEGF is only critical for endothelial cell survival within a defined zone around arteries, where high oxygen and low VEGF levels prevail. However, when animals were exposed to hyperoxia, this zone dramatically expanded, and animals that lacked astrocyte-derived VEGF were more affected. Most noticeably, radial arteries and veins became more susceptible to collapse in mutant animals. These large vessels have been considered to be resistant to hyperoxia due to their maturity and, in the case of arteries, due to their association with vascular smooth muscle cells [25]. However, our results demonstrate that reducing the supply of VEGF can still affect these vessels within the first postnatal week of retinal vasculature development. Why in this instance other sources of VEGF in the retina do not rescue the dying vessels is not clear. Although we found no changes in VEGF isoform ratios in our mutant mice, it is likely that an additional layer of complexity is added by mechanisms that control distribution and bioavailability of VEGF protein. Since our study focuses only on *Vegf* mRNA production and distribution we cannot exclude that the distribution of astrocyte-derived VEGF protein might be different from neuron-derived VEGF protein.

In pupillary membrane vessels, which naturally regress during development, it has been shown that blocking VEGF function accelerates regression [19]. Macrophages can mediate local endothelial cells death [43] and cause the cessation of blood flow, which then leads to the appearance of synchronous apoptosis throughout the entire vessel [19], [44]. In our system, we observed a similar phenomenon, where radial vessels displayed dying cells throughout their entire length and degenerated synchronously within 24 hours of hyperoxia exposure. This is most likely mediated through blood flow interruptions caused by local narrowing of vessel lumens. The higher vulnerability of veins in comparison to arteries could be explained by the lower numbers of smooth muscle cells and pericytes coating the vessels, as it is likely that the transition to VEGF-independence is based on a maturing relationship between endothelial cells and pericytes [26]. Furthermore, *Vegf* mRNA levels were lower along arteries than along veins during hyperoxia exposure (Fig. 4A–C). This explains the relative insensitivity of VEGF deletion in arteries compared to veins.

Vessel destabilization and obliteration are critical processes in the pathogenesis of ROP where supplemental oxygen therapy and lack of growth factors causes vessel loss [45]. IGFBP3 and Epo have both been shown to support endothelial cell survival and mouse strains lacking these factors displayed increased hyperoxia-induced vessel loss [46], [47]. However, to what degree the signalling of these vessel-protective factors involves retinal astrocytes is not well understood and so far they have not found application in the clinic. On the other hand, anti-VEGF therapy has now been applied in ROP cases [48], with the intention to prevent vascular proliferation. Although it is likely that these approaches do not result in a complete deletion of VEGF activity in the retina, our study highlights the important vessel stabilizing function of VEGF in the immature retinal vasculature.

## Materials and Methods

### Mice

All animals were handled in accordance with the UK Animals (Scientific Procedures) Act 1986. The project was approved by the UK Home Office and the UCL Institute of Ophthalmology Ethics Sub-Committee (approval ID PPL 70/6305). Transgenic mice expressing Cre recombinase under the control of promoter segment from the human *GFAP* gene [31] were crossed into homozygous backgrounds of conditional VEGF (*Vegf* ^c/c^) [33] and conditional HIF1α (*Hif1a* ^c/c^) [36]. Analysis was performed on litters containing animals with the Cre transgene and control littermates without it. Litters were exposed with their feeding mothers to hyperoxia (75% Oxygen) as described previously [23].

### PCR

To confirm Cre recombinase activity genomic DNA was isolated from P5 retinas and amplified by PCR using Megamix (Cambio Ltd.) containing 2% DMSO with the following primers: forward CAG GCT TCG GTG GGG TGT GA and reverse GAG CAG GGA TAG GTG GTG GAG ATA. To assess VEGF isoforms reverse-transcribed cDNA obtained from P5 retinas was amplified using Taq polymerase (Promega). The VEGF isoform primer sequences were: forward GAA GTC CCA TGA AGT GAT CCA G and reverse TCA CCG CCT TGG CTT GTC A, as described previously [49]. The primers which were located on exon 3 (forward) and exon 8 (reverse), can amplify all the isoforms of murine VEGF. Different isoforms were identified in a 2.5% agarose gel according to the molecular weight of PCR products.

### Histology

Retinal whole mount preparations were dissected from whole eyes after brief fixation in 2% (w/v) paraformaldehyde (PFA) in phosphate-buffered salt solution (PBS) and then processed for wholemount immunohistochemistry or in situ hybridization as previously described [9]. In some instances, fluorescent anti collagen IV immunostaining was carried out following in situ hybridization to visualize blood vessels. Proliferation was visualized in whole mount preparations that have been stained to visualize vessels with subsequent fixation and HCl treatment prior to anti-BrdU immunohistochemistry, as previously described [9]. For X-gal staining tissue was dissected, fixed in 0.2% glutaraldehyde and processed as previously described [50]. Antibodies used were rabbit anti-Claudin 5 (Invitrogen), rabbit anti-collagen type IV (AbD Serotec), goat anti-collagen type IV (GeneTex), FITC-conjugated isolectin B4 (Vectorlabs), Cy3-conjugated mouse anti-GFAP (Sigma), mouse anti-BrdU [51], rabbit anti-active caspase 3 (Abcam) and secondary Alexa-488 and Alexa-546 antibodies (Molecular Probes).

### Vascular network analysis

Network morphology of the retinal vasculature was analyzed in retinal whole mount preparations from 6 P5 and 5 P10 conditional VEGF litters and in 5 P5 conditional HIF1α litters. We also analyzed 5 further litters after hyperoxia exposure for 5 days at P12 and 5 litters after 1 day of hyperoxia at P8. The surface area of the developing vascular network, width of capillary free spaces and vascular depleted areas in hyperoxia-exposed animals were measured using ImageJ. BrdU positive cells and artery side branches were counted by hand over fixed distances from the retinal centre. Intact and degenerating radial arteries and veins were counted by hand in each retina. Measurements were averaged for control and mutant animals in each litter and normalized against the control values. Statistical analysis was then performed across values obtained from each litter.
